# Application of a Titanium Screw for the Hemorrhage from the Bone Channel during the Lateral Window Technique: A Technical Note

**DOI:** 10.1155/2023/9910646

**Published:** 2023-05-10

**Authors:** Hidekazu Yamamoto, Kazuhiro Matsushita, Hirokazu Takada, Masahiko Suito, Masato Tamura

**Affiliations:** ^1^Kamishihoro Dental Clinic, E4-241, Kamishihoro-cho, Katogun, Hokkaido 080-1408, Japan; ^2^Department of Biochemistry and Molecular Biology, Graduate School of Dental Medicine, Hokkaido University, N13 W7, Kita-ku, Sapporo, Hokkaido 060-8586, Japan; ^3^Stomatognathic Function, Center for Advanced Oral Medicine, Hokkaido University Hospital, N13 W7, Kita-ku, Sapporo, Hokkaido 060-8586, Japan

## Abstract

Pulsatile and profuse hemorrhage occurred in the lateral window technique for implant placement. The surgery was performed in the dental clinic under local anesthesia. The posterior superior alveolar artery was suspected to be the main feeder. Ordinary methods for hemostasis, such as vasoconstrictor-soaked gauze compression, electrocautery hemostasis, absorbable hemostat packing, and bone wax application, were tried. However, strong pulsatile bleeding could not be controlled at all. Complete hemostasis was hardly expected. The idea came up when the titanium screws came into sight. The sterilized screw was always stocked for bone grafting. After visualizing the bleeding point clearly by suction, and the screw was inserted into the bone channel. The bleeding was completely stopped immediately. It may not be a novel method, but is certainly a reliable application of the screw, which is fundamentally the same as arterial catheter embolization.

## 1. Technical Note

**Figure 1:**
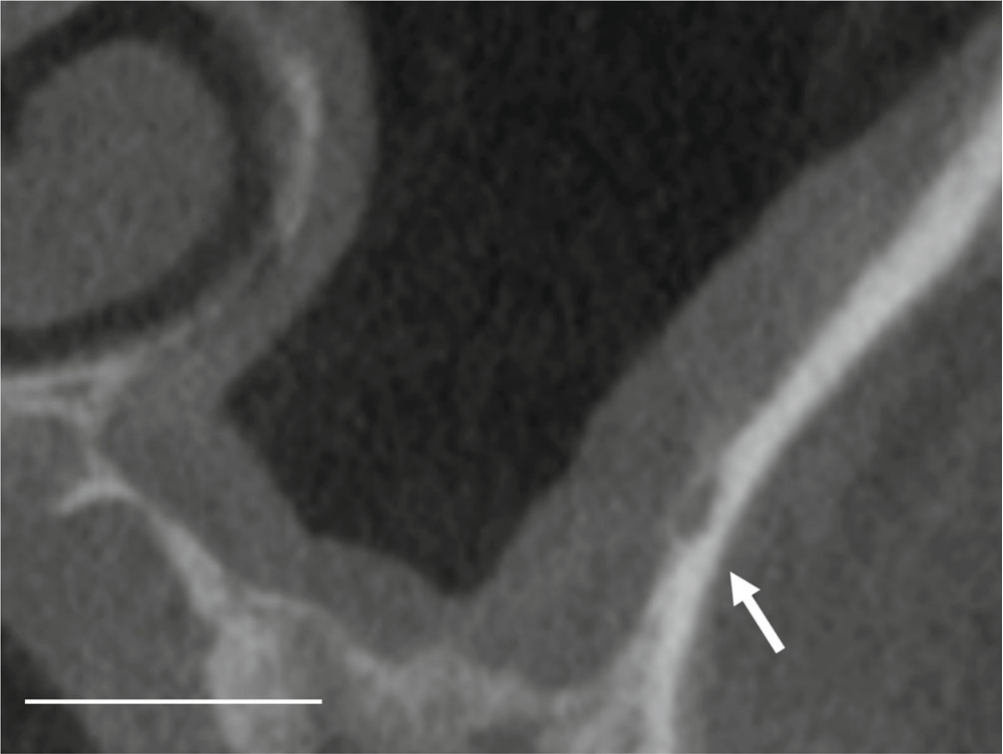
Coronal view of the base of the maxillary sinus in region of tooth #26. The posterior superior alveolar artery (arrow) is observed along the inner aspect of the cortical bone of the sinus wall. (White bar indicates 10 mm).

A computed tomography (CT) scan was performed preoperatively to evaluate the sinus condition and locate the intraosseous vessels (Figure 1). Sinus floor elevation via the lateral approach was conducted using a piezoelectric diamond insert OT5A (Mectron s.p.a., Carasco, Italy) [1, 2]. Sinus antrostomy was safely performed, and membrane elevation was done thereafter. During the elevation, pulsatile and profuse hemorrhage suddenly occurred, seemingly from a relatively large branch and not from small branch ramifications of the artery. A branch located just internal to the wall was suspected to be torn during the detachment of the sinus membrane from the inner aspect of the wall.

The sinus membrane was carefully elevated to avoid further membrane tears, so that space should be prepared for gauze compression. Vasoconstrictor (1 : 80,000) soaked gauze was placed into the space between the membrane and sinus wall. Hemorrhage from the sinus membrane was controlled, but not from the wall. The inner wall of the sinus was repeatedly compressed for 10 minutes. However, hemostasis could not be achieved, and there was no obvious effect with absorbable hemostat [3] or bone wax [4]. Electrocautery was also not effective. For direct visualization and handling, the distal bone edge of the antrostomy was removed until the bone channel of the culprit vessel was clearly identified.

**Figure 2:**
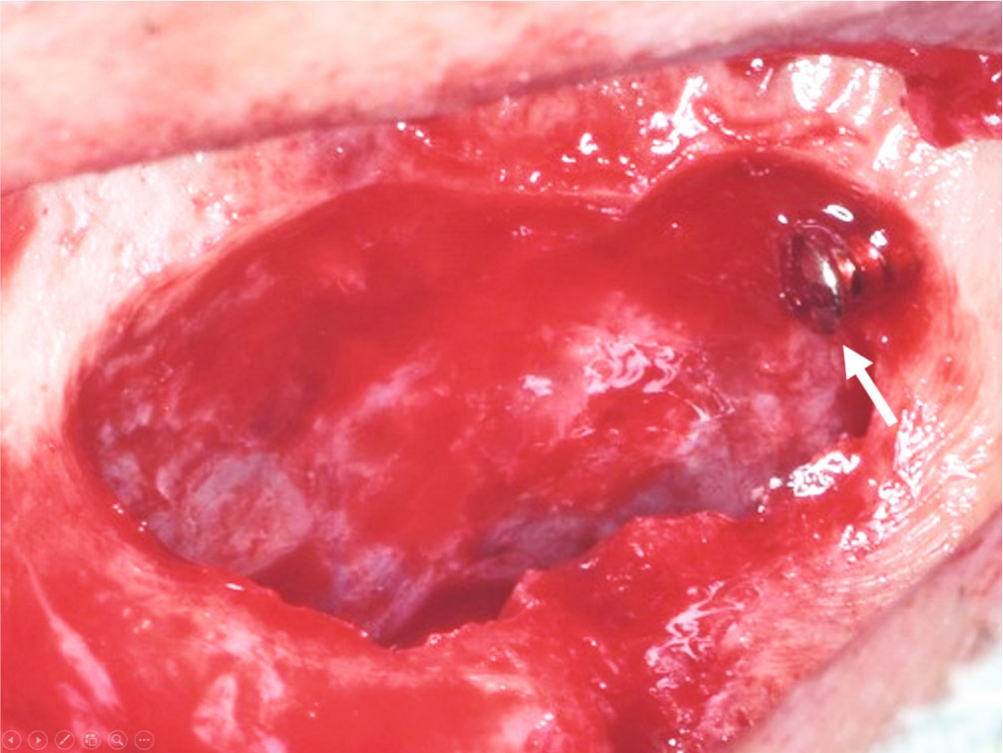
Titanium screw insert for hemostasis. Complete occlusion of the bone channel of the posterior superior alveolar artery achieved by the screw (arrow). The sinus membrane was sufficiently elevated without perforation, and desirable space was created for bone grafting. After achieving hemostasis, bone grafting was performed using Bio-Oss®, as planned.

**Figure 3:**
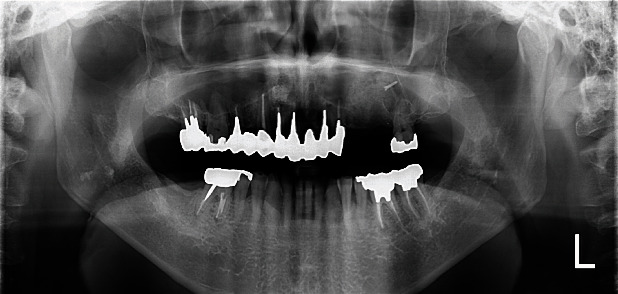
Orthopantograph immediately after the placement of the titanium screw into the bone channel. In the upper region of tooth #27, the titanium screw is seen with the tip facing distal direction. Sufficient volume of bone grafting material is seen, as well.

**Figure 4:**
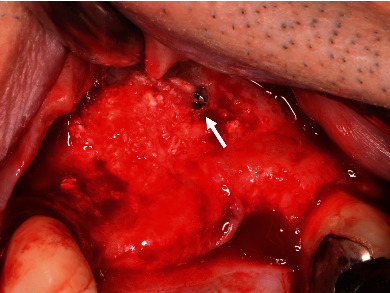
Post-operative condition five months after channel occlusion using the titanium screw. The screw (arrow) is still in place, and sufficient bone augmentation has been achieved. It could easily be removed later, without any complication, followed by implant placement.

It was suspected to be the posterior superior alveolar artery [5, 6]. Firm pressure was applied directly over the channel. The severity of hemorrhage reduced, but complete hemostasis could not be achieved. Then, the idea of local vessel occlusion emerged. A sterile gutta percha point #100 was provisionally inserted proximally into the bone channel as deep as possible, and complete hemostasis was achieved. Convinced that physical occlusion was effective, the gutta percha point was replaced with a titanium screw (Le Forte System screw, diameter: 1.4 mm, length: 6.0 mm; Jeil Medical Corp., Seoul, Korea; Figure 2). Complete and definite hemostasis was achieved. Artificial bone grafting (Bio-Oss®, Geistlich Biomaterials, Wolhusen, Switzerland) was then performed as planned (Figure 3) [7]. Five months later, the screw was removed without any complications (Figure 4), and the implant was placed.

## 2. Discussion

Intra-operative hemorrhage is one of the complications associated with sinus floor elevation [8], even if the pathways of the vessels have been identified pre-operatively using CT. This is partially because the extra-osseous branch of the vessels emerging from the internal aspect of the sinus wall cannot be identified on the plain CT. If hemorrhage occurs, it is usually controlled by applying direct pressure on the bleeding point, electrocautery, bone wax, or crushing the bone channel. However, routine maneuvers are not effective at all if a relatively large arterial branch is injured. Three suppliers (posterior superior alveolar, infraorbital, and the posterior lateral nasal arteries) are the major feeders [9]. We encountered hemorrhage, seemingly from the first one, at the inner surface of the sinus wall and had difficulty in achieving hemostasis, but finally managed it using a novel method. Although this approach is somewhat of a conceit, it is fundamentally the same as arterial embolization and is easy to apply.

As for the re-bleeding during the screw removal procedure, bone screw crest was firmly engaged into the inner surface of the bone channel. Once active hemorrhage is controlled by the insertion of the screw into the bone channel and blood flow gets obstructed, normal process of coagulation is locally initiated. Finally, the bone channel is filled with newly formed bone. There is little chance of re-bleeding when the screw is removed 5 months after sinus floor elevation.

## Data Availability

Data supporting this research article are available from the corresponding author or first author on reasonable request.
